# Application of system dynamics approach in developing health interventions to strengthen health systems to combat obesity: a systematic literature review and critical analysis

**DOI:** 10.1186/s12889-025-22821-1

**Published:** 2025-04-29

**Authors:** Lingyu Zheng, Yan Xue, Xianwen Chen, Carolina Oi Lam Ung, Hao Hu

**Affiliations:** 1 State Key Laboratory of Quality Research in Chinese Medicine, Institute of Chinese Medical Sciences, University of Macau, Macao, China; 2 Centre for Pharmaceutical Regulatory Sciences, University of Macau, Macao, China; 3 Department of Public Health and Medicinal Administration, Faculty of Health Sciences, University of Macau, Macao, China

**Keywords:** Obesity, System dynamics modeling, Health interventions, Health systems

## Abstract

**Background:**

Obesity is an escalating global public health challenge that is expected to impact a significant portion of the world's population in the coming decades. It leads to severe health conditions such as diabetes, cardiovascular diseases, and cancer, imposing significant economic burdens on health systems. Traditional intervention strategies, which emphasize individual lifestyle changes, fail to address the complex, systemic nature of obesity. This study aims to systematically review the application of system dynamics modelling (SDM) in obesity control, focusing on analyzing modelling methodologies and conducting a quality assessment of the included studies.

**Methods:**

Employing a comprehensive systematic literature retrieval, we explored terms pertinent to overweight/obesity and system dynamics across three databases, including PubMed, Web of Science, and Scopus. This search culminated in identifying peer-reviewed studies published from the inception of these databases until July 2024. Quality assessment was used to evaluate the SDM for obesity control. The protocol of this systematic review has been registered on PROSPERO (CRD42024554520).

**Results:**

Thirty studies were identified through a systematic review. These studies primarily focus on the effects of SD approaches, such as individual lifestyle changes, policy interventions within populations, and socio-economic and environmental improvements on obesity control. Among them, eleven studies completed the entire SDM process. Twenty-seven studies presented conceptual models, of which twenty-five developed casual loop diagrams (CLD). Seventeen studies conducted computational system dynamics modelling, with thirteen constructing stock-flow diagrams (SFD). Additionally, fourteen studies performed simulation analyses. These models facilitated multi-level strategies to reduce obesity prevalence.

**Conclusions:**

Using SDM approaches has significant potential to enhance the effectiveness of obesity interventions and optimize resource allocation. Our study into the application of SDM in the design of obesity health interventions revealed its ability to promote multi-level, cross-sectoral cooperation and coordination, thereby enhancing the effectiveness of interventions. Further exploration and optimization of obesity health interventions can significantly advance health systems and welfare.

**Supplementary Information:**

The online version contains supplementary material available at 10.1186/s12889-025-22821-1.

## Background

Obesity is a pervasive and pressing public health issue that poses a significant challenge to health systems worldwide nowadays [1, 2]. It is projected that the global prevalence of obesity will surge from 14% in 2020 to 24% by 2035, affecting nearly two billion adults, adolescents, and children [3]. Obesity serves as a principal driver of numerous diseases, including metabolic disorders and a range of non-communicable diseases such as diabetes, cardiovascular diseases, and cancer, thus imposing a substantial burden on public health infrastructures [4, 5]. Additionally, obesity incurs significant direct medical costs and indirect costs [6–8]. The economic impact of overweight and obesity is expected to grow from 1.96 trillion USD in 2020 to 4 trillion USD by 2035 [4]. This phenomenon is evident in both developed and developing countries, highlighting the universality and severity of the obesity crisis [9].

Traditionally, the strategies for preventing obesity focused principally on changes in the lifestyles of individuals, particularly dietary habits and physical activity levels. This approach ignores the complex and systemic causes of obesity [10]. However, the emergence of the obesity crisis shows a strong relation with socio-economic position, dietary culture, and the food environment. All these factors together have resulted in an unprecedented rise in the cases of obesity both in developed and developing countries, which further complicates the task of responding to this global health problem [11, 12].

In 2019, the Lancet Obesity Commission emphasized the importance of systems science approaches in obesity research [13]. Such an endorsement underscores the need to employ methodologies that can unravel the complexities of obesity, making system dynamics particularly pertinent. System dynamics modeling (SDM), an integral component of systems science, dissects the intricate interdependencies among variables within a system and their resultant nonlinear behaviors [14]. Using simulation to anticipate the outcomes of interventions on complex health dilemmas, SDM provides a distinctive perspective for analyzing and tackling the issue of obesity [15]. These models effectively capture the complexity and multiple factors involved in obesity, showing how it develops over time [16]. In addition, they help simulate both the long-term and delayed effects, making it easier for policymakers to predict the outcomes of different obesity interventions more accurately [17, 18]. Addressing obesity necessitates comprehensive intervention strategies that span individual behavioral modifications, policy development at the societal level, and enhancements to the environmental backdrop [19–21]. SDM facilitates a deeper understanding of cross-level interactions, evaluates the cumulative impacts of diverse interventions, and thus underpins the development of robust, scientifically-backed strategies for comprehensive obesity management [22].

Nevertheless, the current literature lacks a thorough analysis and evaluation of the methodological approaches of SDM in obesity health interventions. Therefore, this study aims to systematically review the application of SDM in developing health interventions to strengthen health systems against obesity through analyzing modeling methodologies. Specifically, this study has three objectives. First, this study aims to explore common methodologies for constructing system dynamics models for obesity. Second, the study aims to analyze the key feedback loops and variables contributing to obesity and examine the simulation outcomes of obesity interventions. Third, the study aims to assess the quality of SDM applications in obesity and propose an evaluation framework for assessing the quality and appropriateness of individual SDM applications. The ultimate goal will be to advance the understanding and practice of SDM in health intervention development and assessment of obesity to improve health systems and prevent and manage obesity.

## Methods

This review was conducted in accordance with the Preferred Reporting Items for Systematic Reviews and Meta-Analyses (PRISMA) 2020 guidelines [23]. Throughout the review process, we systematically identified, summarized, and thoroughly analyzed scholarly papers that develop or apply SDM to prevent and control obesity. The protocol of this systematic review has been registered on PROSPERO (CRD42024554520).

### Search strategy

We conducted searches on electronic databases including PubMed, Web of Science, and Scopus. We used the following keywords in the title, abstract, and keyword fields to ensure the capture of literature closely related to the target theme: (Obesity OR "Weight Loss" OR Overweight OR Adiposity OR obes* OR Bodyweight OR "Body Weight") AND "Systems Analysis" OR "Nonlinear Dynamics" OR "Computer Simulation" OR ((system dynamic OR system dynamics) AND (model* OR simulat*)) OR nonlinear dynamics OR dynamics simulation model OR causal loop OR causal loops OR (stock* AND flow* AND (model* OR simulat*)) OR "system dynamic" OR "system dynamics" OR "system* modeling" OR "system thinking" OR "system science" OR "system approach" OR "system theory"). Additionally, to further enhance the accuracy and completeness of our retrieval, we tailored our search strategies to each database's specific features. Beyond keyword-based retrieval, we employed a "snowballing" technique, identifying more relevant articles through citation and author searches. This method helped us uncover literature that may not have been captured through keyword searches.

### Literature selection

The following review strategies were adopted for this study to be certain that the selection of research literature met established criteria. Initially, the titles and abstracts of all publications retrieved through the searches were independently reviewed by two researchers (LZ and YX). Subsequently, these two researchers thoroughly reviewed the full texts of the articles that passed the initial screening to confirm further whether each article met all eligibility criteria. Specifically, articles had to meet the following conditions to be included in the study. First, the article must be published in a peer-reviewed academic journal to ensure its scholarly quality and reliability. Second, the article must be written in English to facilitate in-depth reading and analysis by the research team. Third, the articles should employ system dynamics modeling techniques such as causal loop diagrams, stock and flow diagrams, or simulation, which are central methodological requirements for this study. Fourth, the article's theme must be closely related to obesity prevention or control, aligning with the core research objectives of the study. Fifth, the article's publication date should be between the establishment date of the databases and July 2024 to ensure the timeliness and completeness of the research. Articles meeting the above criteria were organized and saved into the EndNote reference management software program to facilitate subsequent data extraction and analysis.

### Data extraction

We developed a standardized data extraction form. First, it included general characteristics such as author(s), year, country, setting, population of focus and study aims. Second, it addressed model development, including data sources, involvement of stakeholders, data analysis methods, tools for data analysis and diagramming. Third, it covered the model characteristics and structure, including the SDM methods, diagramming, subsystems, feedback loops, main model variables, stocks and flows. This structured form was designed to facilitate the thorough and systematic extraction of relevant data from the included studies, ensuring consistency and comprehensiveness in capturing critical information necessary for analysis and synthesis.

### Quality assessment

To evaluate the quality of system dynamics modeling and documentation, this study developed nine quality criteria based on three key references [24–26], covering research objectives, assumptions, design, validation, data sources, and result interpretation to ensure that the models effectively capture the complexity and real-world impact of obesity interventions. Scores were assigned to each criterion (0–5), and the total score reflected the overall quality of the study. A detailed description of the assessment process is provided in the Supplementary File (Table S2).

These criteria offer a comprehensive evaluation framework for SDM in obesity interventions. Obesity is a complex public health issue influenced by multiple factors, including lifestyle, community engagement, and socioeconomic conditions [27]. System dynamics models are well-suited to simulate the long-term effects of interventions through dynamic features such as feedback mechanisms and delay effects [28]. Therefore, clarity in research objectives and explicit assumptions ensures that the model accurately reflects the key drivers of obesity and aligns with real-world interventions [29]. Clear model design and structure transparency help researchers understand and apply the model, while validation and data reliability ensure the scientific rigor and applicability of the model in addressing obesity issues [30]. Moreover, the presentation and interpretation of results provide strong support for policymakers in designing targeted and effective obesity strategies [31]. Thus, these criteria not only provide a scientific basis for evaluating system dynamics models in obesity interventions but also ensure their operability and relevance.

## Results

### General characteristics of included literature

**Fig. 1:**
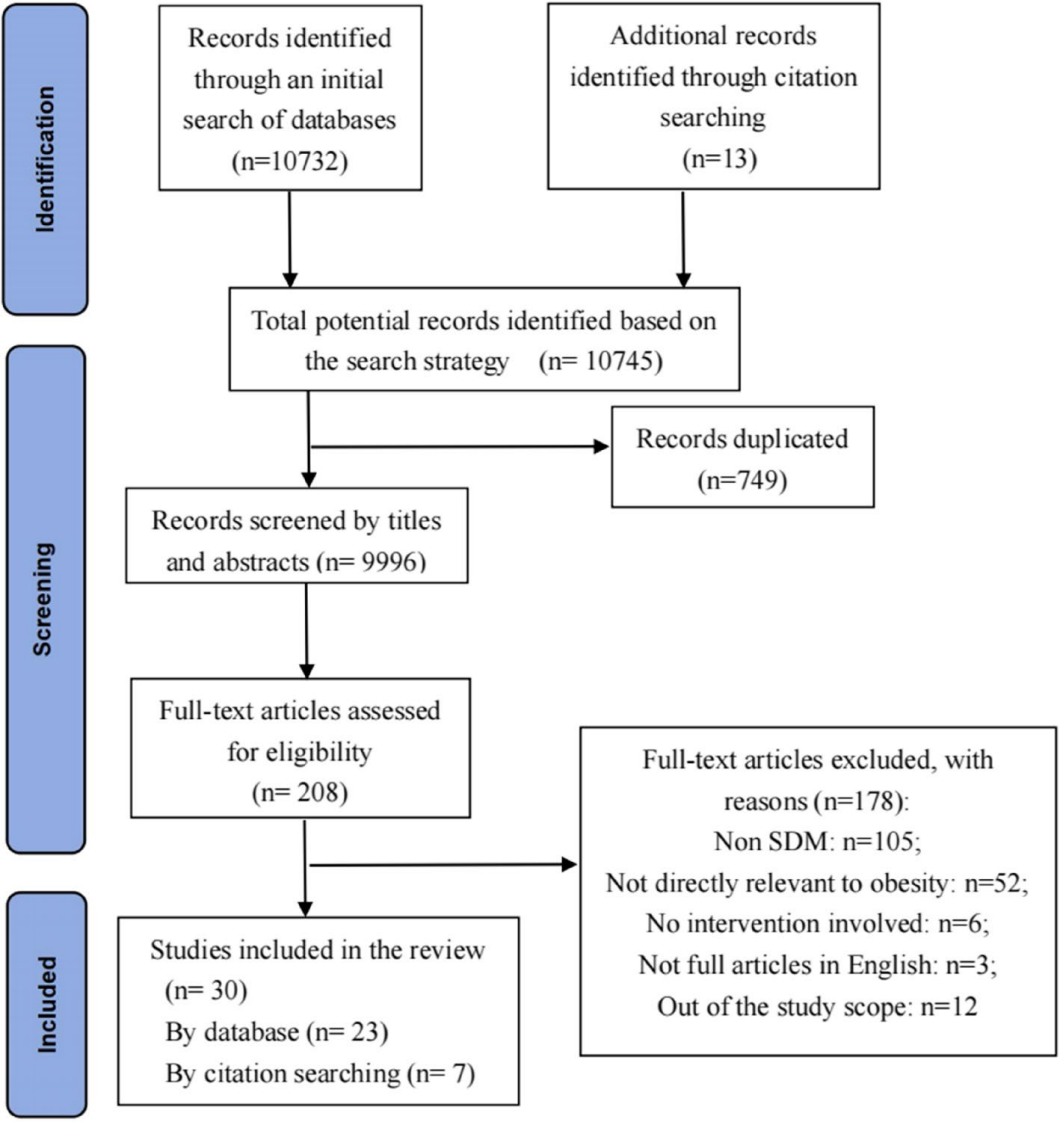
PRISM diagram of identification and selection of studies for the systematic review

Through electronic searches, 10,745 records were identified, from which 749 duplicates were removed, leaving 9,996 records for preliminary screening. In a two-step process for screening the identified records, each record’s title and abstract were first assessed to determine if they met the inclusion criteria. Records whose titles and abstracts did not provide sufficient information for determination were temporarily retained for further review. After the initial screening, the full texts of 208 records were evaluated. During this secondary screening, 178 articles that did not meet the inclusion criteria were excluded, ultimately resulting in 30 studies being included in the review (see Fig. 1).

All 30 selected studies were published between 2011 and 2024 (see Table 1). The majority of these investigations were conducted in developed countries, with 10 studies taking place in the United States [32–41]. The settings of the studies were diverse, encompassing both general and specific locales, including four studies [33, 39, 42, 43] that focused on particular communities, three [44–46] on schools, and one on a household setting [47]. Thirteen studies exclusively examined children [33, 39–41, 43, 44, 46, 48–54], five focused solely on adolescents [44, 45, 55–57], three targeted both children and adolescents [35, 36, 47], three investigated adults [34, 58, 59], and two focused on children and their families [38, 40]. Additionally, four studies addressed broader groups, such as community members [42], adults and youth [37], and the general population [60, 61]. The principal study aims of these studies were to apply system dynamics conceptual modelling to explore the systemic causes of obesity in specific populations within particular contexts and to analyze or predict the effects of obesity interventions through SD computational modelling and simulation.

**Table 1:** General characteristics of the included studies

Ref	Author(s), Year	Country/Region	Setting	Population of focus	Study aims
[48]	Romanenko et al., 2023	31 countries, EU & US	General	School-aged children	To describe the development of a system dynamics model of AdOWOB as part of the EU-funded CO-CREATE project
[55]	Pinzon et al., 2023	Netherland	Three neighbourhoods with a low socioeconomic status in the Amsterdam East city district	10- to 14-year old adolescents	To explore the dynamics behind obesity-related behaviours in adolescents aged 10 to 14 in Amsterdam, incorporating perspectives from researchers, adolescents, and local stakeholders
[32]	Calancie et al., 2023	US	Greenville County, Southern Carolina	Children in Greenville County, especially historically marginalized groups	To promote whole-of-community change that supports childhood obesity prevention through a theory-informed, stakeholder-driven intervention
[44]	Hendricks et al., 2022	South Africa	Schools in the Western Cape province	Adolescents aged 16–18 years	To identify meaningful policy-relevant insights into the drivers of adolescent obesity, as described by the young people themselves in a South African context
[58]	Guariguata et al., 2022	Caribbean, Jamaica	Jamaica, with a focus on non-communicable diseases like diabetes and obesity	Jamaican adults (20 + years)	To model necessary changes in diet and exercise to meet WHO Global Action plan goals and explore interventions for reducing diabetes and obesity
[45]	Savona et al., 2021	Netherlands, Norway, Poland, Portugal, and UK	Schools	Adolescents aged 16–18 years	To identify adolescents' perceptions of obesity drivers in five European countries using Group Model Building (GMB)
[42]	Maitland et al.2021	Australia	Southwest of Sydney CBD, specifically the Campbelltown LGA community	Community members	To build capacity among key leaders and the broader community to apply systems thinking techniques to develop community-led actions that address childhood obesity
[59]	Crielaard et al., 2020	Netherland	Amsterdam	Dutch, Moroccan, and South-Asian Surinamese men and women	To explore the effect of health awareness and norms on group-level BMI through system dynamics models (SDMs)
[56]	Waterlander et al., 2020	Netherland	Amsterdam-East	10–14-year-old adolescents in lower socioeconomic and ethnically diverse groups	To develop and evaluate a dynamic action program targeting childhood overweight and obesity using system dynamics and participatory action research
[33]	Swierad et al., 2020	US	Manhattan’s Chinatown community, New York city	Children in the Chinese American community	To develop a qualitative and socioculturally tailored systems model of childhood obesity within the Chinese American community using group model building (GMB) methodology
[49]	Allender et al., 2019	Australia	Great South Coast region of Victoria	Children within the communities	To apply community-based participatory system dynamics for developing, implementing, and evaluating community efforts to improve children's health
[34]	Chen et al., 2018	US	An ecological study and simulation in the United States	Adults aged 21–65 years	To study country-level dynamics between population weight status and socio-economic distribution in the US and to project impacts of socioeconomic intervention options on obesity prevalence
[50]	Roberts et al., 2019	Australia	New South Wales (NSW), Australia	Children aged 0–17 years	To forecast the impact of various interventions on reducing childhood overweight and obesity in NSW by 5% over 10 years
[35]	Powell et al., 2017	US	Georgia	Children and adolescents aged 18 and under in Georgia	To evaluate the impact of various policy interventions on reducing childhood obesity in Georgia through systems thinking and simulation modelling
[51]	Carrete et al., 2017	Mexico	Urban areas, specifically focusing on Toluca City	Elementary school children aged 9–12 years	To understand how socioecological factors influence children's dietary habits and physical activity, considering the impact of relevant social factors on the development of healthy behaviour patterns among urban Mexican children
[36]	Liu et al., 2016	US	National level, focusing on childhood obesity prevention	Children and adolescents (2–19 years)	To evaluate long-term, dynamic health impacts of taxing sugar-sweetened beverages (SSB) and recycling revenue to support related interventions
[37]	Kuo et al., 2016	US	Los Angeles County	low-income adults and youth	To evaluate the health impact of obesity prevention strategies (physical activity promotion, health marketing, healthy food environments)
[38]	Nelson et al., 2015	US	Urban environment, specifically Milwaukee, Wisconsin	Children and families in Milwaukee	To explore the systemic influences on childhood obesity in Milwaukee and evaluate local intervention strategies
[39]	Brennan et al., 2015	US	49 communities across the United States and Puerto Rico	Children in the communities involved in Healthy Kids Healthy Communities (HKHC) program	To evaluate the use of systems thinking and group model building in understanding and addressing childhood obesity
[52]	Shahid et al., 2015	Canada	Calgary, Canada	4.5–6 years children	Utilized local spatial analysis to evaluate the relationship between childhood obesity and overweight with associated risk factors at the neighborhood level and employed simulation modelling to explore localized interventions, particularly neighborhood walkability, designed to reduce childhood obesity and estimate their potential long-term impact
[40]	Keane et al., 2015	US	Rural Southwest, New Mexico	Children and their families in rural, triethnic (Hispanic, American Indian, non-Hispanic white) community	To identify policy and environmental changes that promote healthy eating, active living, and prevent childhood obesity
[43]	Allender et al., 2015	Australia	A rural Australian community	Community members, focusing on childhood obesity	To identify community perspectives on the causes of obesity using Group Model Building (GMB)
[53]	Zainal Abidin et al., 2014	United Kingdom	General	British children were categorized by gender and three age groups (2–4 years, 5–10 years, and 11–15 years)	To simulate the effects of changes in eating behavior on childhood obesity and assess the time needed to reach the government's obesity prevalence target
[46]	Lan et al., 2014	NA	Elementary schools	Elementary school students	To investigate factors affecting students' BMI values using system dynamics modeling
[54]	Zainal Abidin et al., 2014	United Kingdom	General	English child population aged 2 to 15 years	To compare and determine effective strategies for obesity prevention by improving the consumption of portion size and meal frequency
[41]	Frerichs et al., 2013	US	General community setting	General child population	To construct and parameterize a system dynamics model to assess the social transmission of behaviors affecting childhood obesity and evaluate intervention strategies
[47]	Weimer-Jehle et al., 2012	Germany	Socially disadvantaged families	Children and adolescents from socially disadvantaged backgrounds	To create a qualitative causal-loop model of obesity development and prevention
[60]	Schneider et al., 2011	Germany	General, with a focus on Western industrialized countries	General population with implications for public health policy	To demonstrate the potential of nutrition-ecological modelling for solving complex health issues like obesity
[57]	Pinzon et al., 2024	Netherland	Amsterdam, specifically three ethnically diverse neighborhoods with lower socioeconomic positions in the Amsterdam East district	Adolescents aged 10–14 years	To develop an action program to facilitate systems changes in obesity-related behaviors in adolescents using a participatory system dynamics approach
[61]	Hagenaars et al., 2024	EU & US	High-income countries, focusing on government policy-making environments related to obesity prevention	The general population, particularly those affected by obesity is across high-income countries	To understand why public health policies that target obesogenic environments often fail to gain political support and how policy inertia in obesity prevention can be overcome using methods from complexity and political sciences

*Abbreviations*: *US* The United States, *EU* Europe Union, *UK* United Kingdom, *AdOWOB* Adolescent overweight and obesity, *LGA* Local Government Area, *CBD* Central Business District

### Model development

Model development is crucial for understanding how different data sources, stakeholder involvement, and analysis methods influence the SDM models in obesity interventions. Table 2 presents the data sources included in the studies, the involvement of stakeholders at different stages, and the methods and tools used for data analysis and model construction.

**Table 2:** Model development of the included studies

Ref	Author(s), Year	Data sources for conceptual modeling (primary/secondary)	Data sources for parameter values in computational modeling	Stage of involvement of stakeholders	Data analysis methods and details	Tools for data analysis and diagramming
[48]	Romanenko et al., 2023	Secondary: HBSC dataset (2002–2014) and literature	HBSC dataset (2002–2014) and literature	• Model structure validation • Policy recommendation	Statistical analysis	Stella Architect software
[55]	Pinzon et al., 2023	Primary & secondary: literature, GMB, interviews	NA	Construction of CLD	Qualitative methods	STICK-E for initial map creation, KUMU for relationship mapping, AI for final representation
[32]	Calancie et al., 2023	Primary & secondary: GMB, existing coalition data	NA	• Formulate question • Construction of CLD • Construction of quantitative model • Validation and implementation(scenario)	Primarily qualitative through GMB sessions and analysis of system dynamics	NA
[44]	Hendricks et al., 2022	Primary: GMB & note-taking	NA	Construction and refinement of CLD	GMB, map creating and merging	STICKE
[58]	Guariguata et al., 2022	Primary: stakeholder workshops Secondary: scientific literature	Historical trends of diabetes and obesity, cross-sectional surveys conducted in Jamaica	Conceptual development of the CLD and simulation model	Details not reported	Online Silico App, R with "deSolve" package, and Vensim PLE Software
[45]	Savona et al., 2021	Primary: GMB	NA	During group model building sessions	Group discussions were used to develop CLD	STICK-E
[42]	Maitland et al.2021	Primary: stakeholders’ engagement database, action register, and project management resources such as communication logs Secondary: literature and previous research,	Action register, stakeholder engagement database, gantt chart and communication log	Throughout the project, including the development of the CLD and the implementation of actions	Quantitative data analysis, stakeholder engagement assessment and visual analysis of geographic distribution	STICKE
[59]	Crielaard et al., 2020	Primary: cross-sectional data, survey Secondary: demographic and socioeconomic data; HELIUS study data (Amsterdam's multi-ethnic cohort)	Primary: Cross-sectional data, survey Secondary: HELIUS study data (Amsterdam's multi-ethnic cohort)	NA	Details not reported	NA
[56]	Waterlander et al., 2020	Primary & secondary: literature reviews, observations, PAR groups, and GMB workshops	NA	Throughout the program development and evaluation	Mixed methods approach, including developmental evaluation and systems thinking	NA
[33]	Swierad et al., 2020	Primary: GMB workshops Secondary: literature reviews	Interviews and group discussions, community health data	During the model building workshops	NA	Vensim PLE
[49]	Allender et al., 2019	Primary & secondary: literature reviews, GMB workshops, local health data and community leader consultations	NA	In the design, implementation, and evaluation stages	NA	NA
[34]	Chen et al., 2018	Secondary: literature, cultural norms and community knowledge	2001–2011 Medical Expenditure Panel Survey	NA	Statistical modeling and simulation experiments	SAS, Vensim ® PLE Plus
[50]	Roberts et al., 2019	Primary: Census data, NSW Health Survey Secondary: systematic reviews, expert opinions, program effectiveness reports	NSW health administrative datasets, population projections from the NSW department of planning and environment, survey data and expert knowledge	During model building, specifically through workshops	Participatory approach, scenario testing, sensitivity analysis	iThink® v10
[35]	Powell et al., 2017	Primary & secondary: US Census data, peer-reviewed reports, expert consultations, and state-specific BMI data	Prevalence estimates for bmi categories, policy impact studies, energy balance estimates from peer-reviewed articles, population predictions	Involved in model development and updating	Systems thinking, simulation modeling, trend analysis, and policy intervention impact prediction	NA
[51]	Carrete et al., 2017	Primary: structured interviews	Survey data from students, school nutritional programs and government policies, cultural beliefs and family data	During the data collection and evaluation of interventions	Qualitative analysis based on interviews and secondary data to construct the SDM	STELLA
[36]	Liu et al., 2016	Secondary: literature review	Rudd Centre, BRFSS, NHANES, NSCH	Validation phase	System dynamics modeling, sensitivity analysis, validation tests,	NA
[37]	Kuo et al., 2016	NA	Los Angeles County Department of Public Health, California Health Interview Survey and NHANES (from 1988–1994 to 1999–2004)	Program design and evaluation	PRISM for health impact forecasting	PRISM, qualitative data analysis
[38]	Nelson et al., 2015	Primary: GMB with community members	NA	During GMB sessions	Analysis of themes from GMB sessions	NA
[39]	Brennan et al., 2015	Primary: GMB sessions, participant engagement	NA	During GMB sessions for building CLD	Analysis of CLD generated from GMB sessions	GMB techniques, Vensim for diagram translation
[52]	Shahid et al., 2015	Primary: geographically weighted regression (gwr) results, neighborhood walkability data Secondary: census data	Primary Health Activity Network Timely Information Management (PHANTIM) data, 2005–2008 Statistics Canada for the 2006 Census	NA	GWR for local analysis, simulation modeling for intervention impact	Vensim
[40]	Keane et al., 2015	Primary: GMB sessions, participant engagement	NA	Model building and identification of influences	Analysis of GMB session transcripts and CLD development	Vensim PLE
[43]	Allender et al., 2015	Primary: Group Model Building sessions, community engagement, stakeholder consultations	NA	Model building and refinement	Analysis of CLD and stakeholder input	Vensim
[53]	Zainal Abidin et al., 2014	Secondary: literature review	Health Survey for England (HSE), literature review, and public health data	NA	SD optimization to model eating behavior changes	Vensim
[46]	Lan et al., 2014	Primary & secondary: elementary school health checks, student surveys, and school nutrition education records	Health survey data for children’s bmi values, parental education and socioeconomic status data, physical activity and dietary behavior data, school nutrition education effectiveness data	Model development and analysis	Vensim software for system dynamics modeling and simulation	Vensim
[54]	Zainal Abidin et al., 2014	Primary & secondary: Health Survey for England and expert interviews	Health survey for england (annual secondary data), physical activity data from experts	NA	Simulation modelling using Vensim software, qualitative and quantitative analysis	VensimTM software version 5.10e
[41]	Frerichs et al., 2013	Secondary: literature review and existing research data	NHANES (2009–2010), existing US surveillance data and literature	NA	Sensitivity analysis and scenario testing	Vensim PLE
[47]	Weimer-Jehle et al., 2012	Primary: expert workshops, interdisciplinary research	NA	Model development and validation	Cross-impact balance analysis, qualitative evaluation	Computer-based cross-impact balance analysis tool
[60]	Schneider et al., 2011	Primary: health-related databases, society, economy, and environment databases, interviews with experts Secondary: scientific journals and books, government publications and research reports	NA	During the literature review and model validation process	Qualitative analysis based on literature review	NutriMod for qualitative modeling
[57]	Pinzon et al., 2024	Primary: participatory workshops with target groups Secondary: literature reviews, experts’ knowledge, and local health data	NA	Involved in developing the action program, particularly in identifying mechanisms and leverage points	The analysis involved summarizing action-group workbooks and identifying mechanisms, leverage points, and actions	NA
[61]	Hagenaars et al., 2024	Secondary: a qualitative literature review, focusing on policy change processes	NA	NA	Stock-and-flow analysis	Vensim

*Abbreviations*: *GMB* Group model building, *CLD* Causal loop diagram, *HBSC* Health Behavior in SchoolAged Children, *HELIUS* HEalthy Life in an Urban Setting, *PAR* Participatory Action Research, *NSW* New South Wales, *BRFSS* Behavioral Risk Factor Surveillance System, *NSCH* National Survey of Children’s Health, *NHANES* National Health And Nutrition Examination Survey, *NutriMod* Nutrition-ecological modelling

In the realm of conceptual modelling, the majority of studies predominantly employ a hybrid approach utilizing both primary and secondary data sources. Primary data sources include direct stakeholder engagement [42, 43], Group Model Building (GMB) sessions [38–40, 43–45, 49, 55, 56], stakeholder workshops [47, 58], and interviews [51, 54, 55]. Secondary data sources are primarily literature reviews, with some studies specifically citing established datasets such as the HBSC [48], NSW [50] datasets, and Census data [35, 50, 52]. In contrast, data sources used for deriving parameter values in computational models tend to be quantitative, frequently relying on specific datasets and databases. These include historical trends of diseases, cross-sectional surveys, longitudinal survey, census data, and peer-reviewed publications [34, 36, 37, 52, 53, 58, 60]. Some studies use cohort study data and existing U.S. surveillance data to inform their models [41, 59]. This indicates the important role of quantitative data in accurately predicting the effects of obesity interventions and the impact of policies.

Twenty-three studies involved the participation of relevant stakeholders. In six of these studies [32, 39, 42, 44, 55, 58], stakeholders were engaged in constructing causal loop diagrams (CLD). Six studies [33, 38–40, 43, 45] saw stakeholder participation in GMB sessions. Six studies [35, 41, 46, 47, 50, 57] involved stakeholders in model building and development. Stakeholders participated in model validation in five studies [32, 36, 47, 48, 60], while two studies [37, 56] involved stakeholders in project evaluation. Additionally, one study [49] was associated with model design, another study [51] with data collection, and one study [32] focused on formulating questions. The involvement with diversity underlines the importance of stakeholders throughout the research cycle, from the designing through to evaluation, and further underlines the relevance and applicability of the results.

Data analysis methodologies employed in the studies include: statistical analysis; qualitative analysis (including but not limited to analyses based on the GMB session and interviews); sensitivity analysis; and mixed-methods research. During the research, data analysis, and charting tools to support these analytical techniques will be seriously applied throughout. These range from system dynamics modelling and simulation software, such as Vensim [46], mapping/chart creation software such as STICK-E and KUMU [55], health impact prediction software such as PRISM [37], to analytical software like iThink®, SAS, and R with a "deSolve" package [50, 58]. Moreover, special online applications and software were also used, such as Online Silico App [58], NutriMod [60]. Many studies adopt participatory research approaches, particularly emphasizing the use of GMB techniques and Vensim software for the analysis and development of CLD, alongside deep stakeholder engagement (see Table 2).

### Model characteristics and structure

Model characteristics and structure demonstrate how SDM is applied to represent the dynamic processes in obesity formation and interventions. Table 3 summarizes the SDM methods used, diagramming techniques, subsystems, feedback loops, and the main variables driving the obesity systems.

**Table 3:** Model characteristics and structure of the included studies

Ref	Author(s), Year	SDM methods	Diagramming	Subsystems	Feedback loops	Main model variables	Stocks (if SFD)	Flows (if SFD)
[48]	Romanenko et al., 2023	Conceptual model Computational model Simulation	CLD with SFD	NA	8 RLs	• Computer overuse • Life dissatisfaction • School pressure • Inadequate breakfast • Dieting • Inadequate exercise • Feel low • Feel nervous • Inadequate fruit • Inadequate vegetable	• Adolescent overweight and obesity (AdOWOB) • Feel low	NA
[55]	Pinzon et al., 2023	Conceptual model	CLD	Adolescents' interaction with the food environment Adolescents' engagement with the physical activity environment Influence of the online environment on adolescents Adolescents, parenting, and socioeconomic context interaction Healthcare professionals' engagement with families Transition from childhood to adolescence	31 RLs	• Adolescent dietary habits • Physical activity levels • Sedentary behaviour • Sleep patterns • Social norms • Food environment • Family environment and parental roles • Online environment	NA	NA
[32]	Calancie et al., 2023	Conceptual model	CLD	Tackling food insecurity Empowering communities Advocating for policy and systemic change	7RLs,7BLs	• Policy systems, and environmental (PSE) changes • Resource flows	NA	NA
[44]	Hendricks et al., 2022	Conceptual model	CLD	NA	5 key FBLs	• Physical activity and social media use • Dietary choices • Socio-economic status	NA	NA
[58]	Guariguata et al., 2022	Computational model Simulation	SFD	NA	NA	• Population inflows • Baseline estimates of prediabetes and diabetes prevalence and population mean body mass index (BMI) • Flow (incidence) rates for prediabetes and diabetes onset and prediabetes remission • Age structures • Physical activity estimates • Dietary intake (including sugar-sweetened beverage consumption) • Effect sizes for interventions and risk factors	• Sub-model on aspects of diet • Sub-model to estimate obesity prevalence • Sub-model on physical activity levels • Normoglycemic population • Pre-diabetes population • Diabetes population	• Population growth • New cases of preDM • Recovery from preDM • New cases of DM • Mortality in NGT • Mortality in preDM • Mortality in DM
[45]	Savona et al., 2021	Conceptual model	CLD	NA	Three key feedback loops were identified: • commercial drivers of unhealthy diets • mental health and unhealthy diets • social media use body image and motivation to exercise	NA	NA	NA
[42]	Maitland et al.2021	Conceptual model Computational model	CLD	Stakeholder engagement Community-led action plan formation Formation of working groups Continuous monitoring	NA	four domains: physical activity, education and knowledge, healthy eating, and social factors	NA	NA
[59]	Crielaard et al., 2020	Conceptual model Computational model Simulation	CLD with SFD	NA	2 RLs,1 BL	• Body weight perception • BMI • Individual ideal BMI • Sociocultural ideal BMI • Norm • Discrepancy between BMI and individual ideal BMI	Weight	• Weight gain rate • Weight loss rate
[56]	Waterlander et al., 2020	Conceptual model	CLD	Family School Neighbourhood Healthcare City	NA	• Community environmental factors • Social support systems • Health behaviors	NA	NA
[33]	Swierad et al., 2020	Conceptual model Computational model	CLD with SFD	Traditional social norms influence typical body image perceptions, child-rearing practices, parental academic pressure, and trust in the healthiness of traditional foods The role of grandparents in childcare Limited parental presence at home due to time constraints Children's extensive time spent indoors	3RLs,2BLs	• Traditional social norms • Grandparent responsibility • Parental time at home • Indoor activities	Weight	• Increasing weight • Decreasing weight
[49]	Allender et al., 2019	Conceptual model	NA	Systems norms Financial resources Human resources Social resources Regulations System operations	NA	• Community engagement • Data monitoring systems • Policy and environmental changes	NA	NA
[34]	Chen et al., 2018	Conceptual model Computational model Simulation	CLD	Weight status Employment status Family income levels	NA	• Socioeconomic status (SES) • Body weight status	NA	NA
[50]	Roberts et al., 2019	Conceptual model Computational model Simulation	CLD with SFD	Healthy food environment Parental resources Healthy social norms Physical activity environment Built environment conducive to PA	NA	• Energy intake and expenditure • Intervention reach and adoption	Child population overweight/obesity percentage by age and energy balance (intake and expenditure)	Rate of children becoming overweight or obese
[35]	Powell et al., 2017	Computational model Simulation	NA	No policy change Physical education Recess After-school programs All of the above policies	NA	• Obesity prevalence among children and adolescents • Policy interventions (physical education, recess, and after-school programs that involve physical activity)	NA	NA
[51]	Carrete et al., 2017	Conceptual model Computational model Simulation	SFD	Macrosystem Exosystem Mesosystem Microsystem	NA	• Social factors (family influence, school environment, and governmental policies) • Physical activity (school and community that promote or hinder physical activity among children)	• Macro level influence • Exo level influence • Micro level influence • Meso level influence and percentage of overweight and obesity	Changes in macro/exo/micro/meso level influence per year
[36]	Liu et al., 2016	Conceptual model Computational model Simulation	CLD with SFD	Public health Political Social Economic subsystems	NA	• SSB consumption rates • Revenue from SSB taxation • Allocation of tax revenue for interventions(e.g., constructing parks or subsidizing healthy food) • Population behavior towards SSB consumption and physical activities • The socioeconomic status of the population	• Excise tax revenues • Total budget for promoting a healthy environment • Actual demand fulfilled • Budget assigned for park development • Net increase in parkland area • Budget for subsidies to children and adolescents • Actual energy intake from SSBs among boys • Cumulative reduction in energy intake from SSBs in boys • Energy expenditure from increased physical activity in parks for boys	• Accumulated tax revenue • Funding for creating a healthy environment • Budget for park development • Expenditure on park construction • Increase in park area • Decline in parkland capacity • Funds allocated to subsidy programs • Expenditure on subsidies • Decrease in energy intake among boys • Increase in energy expenditure from activities for boys
[37]	Kuo et al., 2016	Computational model Simulation	NA	Physical activity promotion Health marketing Creation of healthy food environments	NA	• Physical activity levels • Poor eating • Community engagement and acceptance • Nutrition standards in food services	NA	NA
[38]	Nelson et al., 2015	Conceptual model	CLD	Healthy eating policies and environments Partnership and community capacity Social determinants Active living policies and environments Health and health behaviors	NA	• Availability of junk food • Family involvement	NA	NA
[39]	Brennan et al., 2015	Conceptual model	CLD	Active living policies and environments Healthy eating policies and environments Partnership and community capacity Social determinants Health and health behaviors	1 RL and 1 BL (Active Living Policies and Environments Feedback Loop [1 reinforcing loop], Healthy Eating Policies and Environments Feedback Loop [1 balance loop])	Community partnerships	NA	NA
[52]	Shahid et al., 2015	Conceptual model Computational model Simulation	CLD and SFD	NA	NA	• Total children • Overweight children • Obese children • Normalized overweight children • Normalized obese children • Immigrant population • Education • Median census family income • Proximity to fast food restaurants • Proximity to parks • Walkscore • Pathway length	Overweight, obese and healthy-weight children	• Healthy to overweight • overweight to obese
[40]	Keane et al., 2015	Conceptual model	CLD	Healthy eating policies and environments Active living policies and environments Health and health behaviors Partnership and community capacity Social determinants	NA	• Access to healthy food • Physical activity opportunities • Community engagement	NA	NA
[43]	Allender et al., 2015	Conceptual model	CLD	Social influences Fast food and junk food Participation in sport General physical activity	4RLs,2BLs	• Physical activity levels • Marketing effects on children • Water quality	NA	NA
[53]	Zainal Abidin et al., 2014	Conceptual model Computational model Simulation	CLD with SFD	Food intake Energy expenditure Physical measurement BMI impact	3RLs,1BL	• Energy intake • Energy expenditure	• Average weight • Average height • Prevalence of obesity	• Change in average weight per year • Change in average height per year • Change in prevalence of obesity per year
[46]	Lan et al., 2014	Conceptual model Computational model Simulation	CLD and SFD	Diet-associated parenting behaviors and students’BMI values The effectiveness of the implementation of school nutrition education and students' health concepts Students’experience of being ridiculed for their body shapes and students' high-calorie diets	4RLs • Diet • Students' self-body perception • Health education • Social influences on BMI	• Students' dietary behaviors • Parental influence on diet	• Students' perceptions of health • Obesity levels • Nutrition education	NA
[54]	Zainal Abidin et al., 2014	Conceptual model Computational model Simulation	CLD with SFD	Food intake Energy expenditure Physical measurement	3 RLs,1 BL	• Average of fat portion size from school/home/outside • Meal frequency • Energy intake • Energy expenditure • Average daily energy balance • Changes in average weight and BMI	• Average weight • Average height • Prevalence of obesity	• Changes in av. + weight per year • Changes in av. height per year • Changes in the prevalence of obesity
[41]	Frerichs et al., 2013	Conceptual model Computational model Simulation	CLD with SFD	NA	2RLs	• Health status (normal weight, overweight, obese) • Social transmission rates (adult-to-adult, adult-to-child, child-to-child) • Intervention impacts (prevention and treatment for both adults and children)	Populations of normal weight, overweight, and obese children and adults	• Normal weight adult/child weight gain rate • Overweight adult/child weight loss/gain rate • Obese adult/child weight loss rate
[47]	Weimer-Jehle et al., 2012	Conceptual model	CLD	Individual Familial Societal level	NA	• Personal context factors (such as physical inactivity, genetic predisposition, family structure) • Societal context factors (like media influence and food availability) • Dependent factors directly influencing energy balance (including physical activity, eating habits, stress levels)	NA	NA
[60]	Schneider et al., 2011	Conceptual model	CLD	Health Environment Economy Societal factors	The study does not specify an exact number of feedback loops in the text; however, it emphasizes the existence of multiple feedback loops inherent in the complex interplay of factors contributing to overweight and obesity	• Socio-economic factors • Food supply • Lifestyle factors • Mental factors	NA	NA
[57]	Pinzon et al., 2024	Conceptual model	CLD	Adolescents' interaction with the food environment Adolescents' engagement with the physical activity environment Influence of the online environment on adolescents Adolescents, parenting, and socioeconomic context interaction Healthcare professionals' engagement with families Transition from childhood to adolescence	31FBLs	121	NA	NA
[61]	Hagenaars et al., 2024	Conceptual model Computational model	CLD with SFD	Current policy inertia in addressing obesogenic environments Leverage points for breaking free from inertia System elements required to lock in policy changes	30FBLs	NA	• Deployment of real-world obesity evidence as part of institutional design • Policy image claim of commercial interests • Obesity policy integration in relevant policy subsystems • Community-based system dynamics capacity focused on obesity policy • Reciprocity between health and other public sectors • Policy image claim of the biomedical sector • Incremental upstream obesity policies targeting the obesogenic system • Boundary spanning capacity of policy brokers	• Upstream obesity policy punctuation • More incremental policies • Policy broker strengthening • Changing use of evidence • More capacity • Change in policy subsystem • More reciprocity

*Abbreviations*: *CLD* Causal loop diagram, *SFD* Stock- flow diagram, *PA* Physical activity, *SSB* Sugar-sweetened beverages, *preDM* prediabetes, *DM* Diabetes, *NGT* Normoglycemia tolerance, *CVD* Cardiovascular disease

The process of system dynamics modeling is typically divided into three stages: conceptual model, computational model, and simulation [62]. In the conceptual model stage, the primary focus is to identify and analyze the causal relationships between variables in the system by building causal loop diagrams (CLD). This stage focuses on qualitative description and theory building. During the computational model stage, the causal relationships in the conceptual model are turned into mathematical formulas, creating stock and flow diagrams (SFD). This step changes the qualitative model into a quantitative one, allowing for more accurate analysis and predictions. At the simulation stage, the computational model is used to simulate the system's evolution, test different scenarios, and predict the future state of the system [63].

Among the thirty studies included, eleven studies completed the full process of system dynamics modeling [34, 36, 41, 46, 48, 50–54, 59]. Of these studies, twenty-seven papers presented conceptual models, with twenty-five studies constructing causal loop diagrams (CLD). Additionally, seventeen studies conducted research on computational system dynamics models, and thirteen of them built stock and flow diagrams (SFD). Furthermore, fourteen studies conducted simulations to test the effectiveness of obesity interventions at different levels.

Subsystems encompass a wide array of factors, primarily including individual behaviors and familial influences, psychological and cultural educational elements, health policies and community engagement, as well as environmental and socioeconomic factors. At the level of individual behaviors and familial impacts, the focus primarily revolves around health and health-related behaviors, partnership dynamics [38–40], and interactions between adolescents and their parents [55]. Regarding psychological and cultural educational factors, they include mental health, unhealthy dietary habits, body image, motivational aspects of physical exercise, traditional societal norms [45], and school-based nutritional education [48]. This layer of subsystems provides a more profound understanding of the potential social and psychological factors contributing to obesity. Concurrently, in the realm of community involvement and policy intervention, the emphasis lies on community empowerment, advocacy for policy and systemic changes [32], and policies promoting healthy diets and active lifestyles [38–40]. In terms of environmental and socioeconomic factors, some studies focus on the food environment [32, 37, 55, 57], physical activity settings [35, 43, 50, 53–55, 57], online environments [55, 57], fiscal and human resources [49], healthcare, and urban contexts [56], all illustrating how dynamics factors influence individual health choices and the prevalence of obesity (see Table 3).

### Main variables and feedback loops driving obesity

A complicated interaction of lifestyle, psychological, and socioeconomic factors drives the increasing prevalence of obesity. Several studies have explored these dynamics using system dynamics models. Eight feedback loops connecting adolescent obesity with lifestyle habits, psychological conditions, and dietary behaviors were identified by Romanenko et al*.* [48], emphasizing how insufficient physical activity and an unhealthy eating pattern contribute to obesity, which in turn aggravates these behaviors and elevates psychological stress. Hendricks et al*.* [44] went further to identify five important feedback loops within South African adolescents, with emphasis on physical activity, unhealthy food intake, social media, and socio-economic status, pointing toward the multifactorial nature of obesity. Savona et al*.* [45] used the GMB to generate three feedback loops. These loops connected food advertising, mental health, and the influence of social media on adolescent obesity. The study covered five European countries and suggested a preference for multi-level prevention strategies. Swierad et al*.* [33] studied childhood obesity in Manhattan’s Chinatown. They explored how sociocultural traditions, family impact, and time spent indoors are interrelated. Their findings highlight the need for culturally sensitive interventions.

Other studies further illustrate the community and education factors contributing to obesity. Nelson et al*.* [38] and Brennan et al*.* [39] focused on how community environments contribute to childhood obesity, emphasizing factors like lack of access to healthy food, limited physical activity opportunities, and socioeconomic challenges within communities. Keane et al*.* [40] identified 27 factors affecting obesity by focusing on the impact of community cultural pride and youth participation in healthy behaviors. In another study, Lan et al*.* [46] made use of system dynamics modeling to investigate how personal lifestyle, family influence, school education, and peer interaction affect children's BMI, presenting health concepts and education as having an important influence on the change of children's BMI. Moreover, Carrete et al*.* [51] examined how community and school education impact childhood obesity, highlighting the lack of sports facilities in communities and insufficient healthy food education in schools (see Table 3).

### Simulated obesity interventions and outcomes

System Dynamics (SD) models are practical tools for simulating obesity interventions, highlighting the multidimensional and interdisciplinary approaches required to address obesity [64]. These interventions range from lifestyle changes to community and government initiatives aiming to reduce obesity rates and improve health systems.

Lifestyle and community-based interventions have proven to be effective in reducing obesity rates. Romanenko et al*.* [48] predicted more than 8% reduction in adolescent obesity across Europe by 2026 through targeted measures such as increasing physical activity, fruit intake, reducing life dissatisfaction, and alleviating academic stress. Calancie et al*.* [32] employed a community-driven strategy, enhancing nutrition programs, food security coordination, and community empowerment, significantly improving public health. Guariguata et al*.* [58] focused on the Caribbean, where combined upstream (dietary improvements) and downstream (targeting high-risk populations) interventions were projected to reduce obesity by 3.4% by 2050. Shahid and Bertazzon [52] explored the impact of neighborhood walkability on childhood obesity in Calgary, finding that increasing walkability scores could noticeably decrease obesity rates. Abidin et al*.* [53, 54] used SDM to simulate the effects of dietary behavior changes on childhood obesity in the UK, showing that while interventions could reduce obesity rates, a longer timeframe is needed to meet governmental targets.

Economic and public health interventions have also demonstrated significant potential in addressing obesity. Chen et al*.* [34] found that a 16.6% reduction in obesity could be done through improving income mobility and increasing employment opportunities. Roberts et al*.* [50] have predicted that a close to 5% reduction in obesity rates by 2025 would be reached by enhanced public health programs and improved environmental facilities, based on nine simulations of childhood obesity-targeted interventions. In another study, Crielaard et al*.* [59] on health awareness and social norms found that health awareness could bring modest weight reductions, but adding social norms greatly improved outcomes, especially for men. Liu et al*.* [36] examined the imposition of a tax on sugary drinks and the recycling of its revenue to fund public health programs, showing that such policies do reduce the rate of obesity.

Comprehensively, multifaceted interventions that involve cooperation between government, community, and families are crucial for effective obesity reduction. For instance, Powell et al*.* [35] were able to demonstrate that instituting a suite of policies at the same time could reduce the rate of obesity from 18 to 3%. In accordance with Carrete et al*.* [51], family and government cooperation will be able to substantially lower childhood obesity rates with a combined approach over 25 years. Kuo et al*.* [37] used the PRISM model with comprehensive strategies focusing on healthy eating, active lifestyles, and health marketing to predict dramatic reductions in obesity and low physical activity rates by 2040. Frerichs et al*.* [41] showed, with a model of social transmission between adults and children, that reducing social transmission would directly lead to reductions in childhood obesity rates.

These interventions not only target individual behavioral changes but also involve structural adjustments at the social and environmental levels. By simulating the implementation effects and long-term impacts of various strategies, SDM helps researchers optimize intervention effectiveness and predict their comprehensive impact on public health, supporting the development of more effective obesity prevention and intervention strategies [65].

### SDM quality assessment report

According to the quality assessment results presented in Table 4, the overall quality scores of the included system dynamics modeling (SDM) studies range from 29 to 37 points, with an average score of 32.6 points (out of a maximum of 45). In the various quality criteria, most studies excelled in the clarity of model design and structure, with an average score of 4.2; in the detailed description of the modeling process, the average score was 4.1. However, the comprehensiveness of model validation received the lowest score of 1.1. Regarding the clarity of research objectives and purpose, explicitness and appropriateness of model assumptions, and clarity and reliability of data sources, most studies scored between 3.6 and 4.0.

**Table 4:** The quality assessment scores of included system dynamics modelling studies

Ref	Author(s), Year	Clarity of research objectives and purpose	Explicitness and appropriateness of model assumption	Clarity of model design and structure	Detailed description of modeling process	Comprehensiveness of model validation and verification	Clarity and interpretability of model visualization	Clarity and Reliability of Data Sources	Present clear model output and results	Results interpreted and discussed in context	Total scores	Average scores
[48]	Romanenko et al., 2023	4	4	4	5	0	3	4	4	4	32	3.6
[55]	Pinzon et al., 2023	4	4	4	4	0	4	4	4	5	33	3.7
[32]	Calancie et al., 2023	4	3	4	4	0	4	4	4	3	30	3.3
[44]	Hendricks et al., 2022	4	3	4	4	0	4	3	4	4	30	3.3
[58]	Guariguata et al., 2022	4	4	4	5	3	4	4	4	4	36	4
[45]	Savona et al., 2021	4	4	4	4	0	4	3	4	3	30	3.3
[42]	Maitland et al.2021	4	4	4	5	0	5	4	4	5	35	3.9
[59]	Crielaard et al., 2020	4	3	4	5	3	4	5	4	5	37	4.1
[56]	Waterlander et al., 2020	4	4	4	4	0	3	4	4	3	30	33
[33]	Swierad et al., 2020	5	4	4	5	0	4	4	4	5	35	3.9
[49]	Allender et al., 2019	4	3	3	3	0	0	4	3	4	24	2.7
[34]	Chen et al., 2018	5	4	4	4	3	3	5	4	5	37	4.1
[50]	Roberts et al., 2018	4	4	5	5	4	5	4	4	4	39	4.3
[35]	Powell et al., 2017	3	3	3	3	0	2	3	3	4	24	3.7
[51]	Carrete et al., 2017	4	4	4	4	3	4	3	4	4	34	3.8
[36]	Liu et al., 2016	4	4	5	4	3	5	4	4	4	37	4.1
[37]	Kuo et al., 2016	4	3	4	3	3	3	4	3	4	31	3.4
[38]	Nelson et al., 2015	4	3	4	4	0	4	3	3	4	29	3.2
[39]	Brennan et al., 2015	4	3	4	4	0	3	3	4	4	29	3.2
[52]	Shahid et al., 2015	4	4	4	4	0	5	4	4	4	33	3.7
[40]	Keane et al., 2015	3	4	4	4	0	4	3	3	4	29	3.2
[43]	Allender et al., 2015	4	3	5	4	0	5	3	3	4	31	3.4
[53]	Zainal Abidin et al., 2014	4	4	5	4	3	5	4	4	4	37	4.1
[46]	Lan et al., 2014	4	3	5	4	0	4	4	4	3	31	3.4
[54]	Zainal Abidin et al., 2014	4	3	5	4	3	5	4	4	4	36	4
[41]	Frerichs et al., 2013	4	4	5	4	3	5	4	4	4	37	4.1
[47]	Weimer-Jehle et al., 2012	4	4	4	4	1	4	3	3	4	31	3.4
[60]	Schneider et al., 2011	4	4	4	4	0	3	3	4	4	30	3.3
[57]	Pinzon et al., 2024	4	4	5	5	0	4	4	4	4	34	3.8
[61]	Hagenaars et al., 2024	5	4	5	4	0	5	4	4	5	36	4
	Total scores	121	109	127	124	32	117	112	113	122	977	
	Average scores	4	3.6	4.2	4.1	1.1	3.9	3.7	3.8	4.1	32.6	

## Discussion

This study systematically reviewed 30 papers that utilized SDM for obesity, focusing on the factors influencing obesity and the intervention measures. First, these SDM studies make it possible to dive into multifactorial reasons and complex interactions behind obesity. Most of these studies reflected the feedback loops of individual behaviors, socio-economic factors, and community influences considerations put together to deepen the complexity of the obesity trait. For instance, unhealthy dietary habits and insufficient physical activity not only directly lead to weight gain but also exacerbate obesity through their impact on mental health and social behaviors [48]. Second, this review explores how SDM can be used dynamically to address the obesity issue. System dynamics models can simulate the long-term effects of different intervention strategies that could show the potential effectiveness of multi-level and multifactorial interventions [66]. For example, they can test how well dietary practice, levels of physical activity, or policy measures work across varying socio-economic contexts and, therefore, be used to guide more precise and effective public health interventions [36, 37, 48].

However, it must be noted that SDM is a context-specific approach that needs to consider contextual factors carefully. In this review, several studies indicate that socio-economic and cultural differences in different contexts significantly impact the effectiveness of obesity interventions. For example, Romanenko et al. [48] found that public health infrastructure and education levels in different European countries influenced the effectiveness of interventions, while the studies by Hendricks et al. [44] and Savona et al. [45] showed different driving factors behind adolescent obesity in South Africa and Europe. Liu et al. [36] studied the policy of taxing sugary drinks and recycling the revenue for public health programs, but the effectiveness of such policies may vary depending on regional economic conditions and the strength of policy support. In resource-limited areas, these policies may face implementation challenges, so it is necessary to adjust the policy parameters in the SDM model based on the specific economic and social environment of the region to achieve the best results. These contextual differences emphasize the need for adjustments in SDM when implemented in different regions to fit the local socio-cultural and economic characteristics.

Regarding modeling research methods, conceptual models, visually represent the complex interactions within the obesity system. These models are crucial for identifying key intervention points and facilitating stakeholder collaboration, particularly in diverse socioeconomic and cultural contexts. For instance, the study of Swierad et al. [33] illustrated how reinforcing feedback loops in social norms, marketing of foods, and family configurations reinforce obesity. Computational models enable researchers to simulate and predict the long-term effects of specific interventions, such as economic policies or public health strategies [22]. Studies by Chen et al*.* [34] and Liu et al*.* [36] found that interventions in economics, such as improving income mobility and taxing sugar-sweetened beverages, could result in significant reductions in the rate of obesity. These models support policymakers in making decisions regarding the potential policy impacts and optimization of resource allocation through scenario simulations. The qualitative and quantitative methods integrated into SDM provide the systematic framework to understand obesity, which can help develop more relevant and effective public health interventions [67].

Most are, however, heavily reliant on secondary data sources from literature reviews and national health datasets that do not always reflect an accurate picture, hence their limitations in real-time conditions at the local and current levels. It is also found that most of the proposed models have not passed through proper calibration or validation with real-world data, which raises some concerns regarding the reliability of model outcomes once applied in practical settings [30]. Therefore, it is necessary for researchers to follow SDM research processes rigorously to ensure the validity of SDM.

However, as identified from studies such as Hendricks et al*.* [44] and Pinzon et al*.* [55], stakeholder involvement typically remains partial to some phases, mainly during the initial construction of CLDs, and does not necessarily involve continuous engagement right through the modelling and validation stages. This might limit the models in capturing evolving system dynamics or reflecting multiple stages of stakeholder perception in intervention planning. While GMB sessions stimulate group collaboration, most research, such as Nelson et al*.* [38] and Brennan et al*.* [39], have utilized single-session GMB processes devoid of follow-up iterations to enhance and refine models. This single session reduces the chances of dynamic interaction and incremental model improvement. Further research will have to consider iterative GMB, involving stakeholders at various stages in the development and validation of the model. This is how adaptive and more precise models and more effective policy interventions would be possible.

At present, research on obesity intervention utilizing SDM predominantly emphasizes behavioral modifications and socio-economic determinants. Nevertheless, the integration of clinical services—including nutritional counseling, behavioral therapy, pharmacotherapy, and surgical interventions—remains insufficiently represented within these models. Integrating clinical interventions into SDM can provide a more comprehensive understanding of obesity management [68]. By combining real-world clinical services with SDM, researchers can simulate personalized treatment plans and predict their long-term outcomes[69, 70], thereby developing more targeted strategies for obesity prevention and treatment, especially in addressing comorbidities like diabetes and cardiovascular diseases[58, 71].

With SDM applications continuing to expand into public health, the development of uniform scoring systems will further enable model comparison capabilities and ensure the validity of research findings [72]. Future research should further explore how clinical services can be integrated with community interventions, considering their application across different socio-economic conditions and cultural backgrounds. For example, in low-income areas, more attention may need to be given to resource availability and community involvement, whereas in high-income areas, the focus might be on lifestyle changes and health education. Through such adjustments, SDM can more effectively address the needs of obesity interventions across different contexts. Moreover, there is a need for future studies to focus on the long-term impact of clinical interventions in preventing obesity relapse and cost-effectiveness as a result of combining clinical services with community-based obesity prevention interventions. In fact, other studies will also be able to establish how such improvement of accessibility and quality of clinical services in either an underserved or rural area impacts obesity prevention strategies [73]. Through these investigations, the practical application of future SDM will be more closely aligned with real-world health systems, providing more robust support for developing targeted obesity interventions[74, 75].

To our knowledge, this is the first systematic review focusing on applying SDM to explore obesity, particularly in revealing the complex drivers of obesity and analyzing the long-term effects of obesity interventions. This review utilized a systematic search methodology covering multiple databases and strictly adhered to explicit inclusion and exclusion criteria. Our analysis of existing models, especially in applying multi-level intervention frameworks, lays a theoretical foundation for future research in the public health domain using SDM. However, there are certain limitations to this review. Firstly, we limited our analysis to studies published in English, which may have resulted in the exclusion of important non-English research and thus affected the comprehensiveness of our findings. Secondly, the heterogeneity among the studies included presented challenges in assigning a consistent score to the models. This variability may have led to significant discrepancies in quality during the model evaluation process, ultimately restricting our overall assessment of model validity. Lastly, due to the diversity in the studied SDMs, we were unable to establish a systematic framework connecting the contextual factors with model components. Future research in this area is highly encouraged.

## Conclusion

The SDM approach has shown notable effectiveness in the development and analysis of obesity health interventions, particularly in reinforcing health systems. Our comprehensive examination of SDM's application in obesity prevention and control strategies highlights its ability to foster integration at the health system level and promote inter-sectoral collaboration. Furthermore, the introduction of SDM allows policymakers to examine the diverse factors influencing obesity from the initial stages of strategy design. These factors encompass lifestyle choices, community engagement, and socio-economic conditions. Such thorough consideration is crucial for optimizing resource allocation while enhancing the flexibility and adaptability of interventions. Accordingly, future research should continue to utilize SDM to improve model accuracy and the practical effectiveness of interventions, ultimately supporting the optimization of health systems and reducing the prevalence of obesity and its associated health complications.

## Supplementary Information

Supplementary Material 1.

Supplementary Material 2.

## Data Availability

Data will be available upon request of the corresponding author.

## Abbreviations

SDM: System dynamics modeling
SD: System dynamics
CLD: Casual loop diagram
FBL: Feedback loop
SFD: Stock and flow diagram
GMB: Group Model Building
HBSC: Health Behavior in School-Aged Children
NSW: New South Wales
USD: United States Dollar
PRISMA: Preferred Reporting Items of Systematic Reviews and Meta-Analysis
