# Supplementary material for: Application of system dynamics approach in developing health interventions to strengthen health systems to combat obesity: a systematic literature review and critical analysis
Source: BMC Public Health. 2025 Apr 29;25:1580. doi: 10.1186/s12889-025-22821-1 (PMC12039072; doi:10.1186/s12889-025-22821-1)
Supplement: Supplementary file 1 — Supplementary Material 1. [file 12889_2025_22821_MOESM1_ESM.docx]

**Supplementary File Table S1:** **Search strategy**

**PubMed (n=5376)**

| **No.** | **Key words (subject headings or free terms)** |
| --- | --- |
| #1 | Obesity[MeSH] |
| #2 | “Weight Loss”[MeSH] |
| #3 | Overweight[MeSH] |
| #4 | obes* OR adiposity OR overweight OR bodyweight OR "body weight" |
| #5 | #1 OR #2 OR #3 OR #4 |
| #6 | Systems Analysis[MeSH] |
| #7 | Nonlinear Dynamics[MeSH] |
| #8 | Computer Simulation[MeSH] |
| #9 | ((system dynamic OR system dynamics) AND (model* OR simulat*)) OR nonlinear dynamics OR dynamics simulation model OR causal loop OR causal loops OR (stock* AND flow* AND (model* OR simulat*)) OR “system dynamic” OR “system dynamics” OR “system* modeling” OR “system thinking” OR “system science” OR “system approach” OR “system theory” |
| #10 | #6 OR #7 OR #8 OR #9 |
| #11 | #5 AND # 10 |

**Web of Science (n=2057)**

| **Concept** | **Queries** |
| --- | --- |
| Obesity | obesity OR obes* OR overweight OR “over weight” OR “body weight” OR adiposity |
|  | AND |
| System Dynamics | ((system dynamic OR system dynamics) AND (model* OR simulat*)) OR nonlinear dynamics OR dynamics simulation model OR causal loop OR causal loops OR (stock* AND flow* AND (model* OR simulat*)) OR “system dynamic” OR “system dynamics” OR “system* modeling” OR “system thinking” OR “system science” OR “system approach” OR “system theory” |

**Scopus (n=3299)**

| **Concept** | **Queries** |
| --- | --- |
| Obesity | obesity OR obes* OR overweight OR “over weight” OR “body weight” OR adiposity |
|  | AND |
| System Dynamics | ((system dynamic OR system dynamics) AND (model* OR simulat*)) OR nonlinear dynamics OR dynamics simulation model OR causal loop OR causal loops OR (stock* AND flow* AND (model* OR simulat*)) OR “system dynamic” OR “system dynamics” OR “system* modeling” OR “system thinking” OR “system science” OR “system approach” OR “system theory” |
