# Supplementary material for: Application of system dynamics approach in developing health interventions to strengthen health systems to combat obesity: a systematic literature review and critical analysis
Source: BMC Public Health. 2025 Apr 29;25:1580. doi: 10.1186/s12889-025-22821-1 (PMC12039072; doi:10.1186/s12889-025-22821-1)
Supplement: Supplementary file 2 — Supplementary Material 2. [file 12889_2025_22821_MOESM2_ESM.docx]

**Supplementary File Table S2: Quality criteria for system dynamics models**

| **Quality criteria** | 0 | 1 | 2 | 3 | 4 | 5 |
| --- | --- | --- | --- | --- | --- | --- |
| 1. Clarity of research objectives and purpose | No mention of research objectives or purpose. | Objectives are vague and lack direct relevance to system dynamics modeling. | Objectives are partially defined but lack specific application context. | Objectives are clear and linked to SD methodology, but no mention of addressing critical gaps. | Objectives are specific, explaining how the model addresses practical or theoretical issues. | Objectives are innovative, proposing new methodological frameworks or cross-domain applications.[1] |
| 1. Explicitness and appropriateness of model assumption | No mention at all. | Briefly mentions assumptions without linking to SD principles. | Describes some assumptions (e.g., time horizon, variable relationships) without defined boundaries or hierarchies. | Full description of assumptions, including subsystems, feedback loops or stock-flow relationships but lacks validation. | Clear assumptions based on literature/empirical data, with explanations of their impact on model behavior. | Clear description of the model's structure, outputs, boundaries (temporal and conceptual), aggregation level, parameter sources, initial values, decision-making processes, exogenous variables, data sources, and overall rationale.[2] |
| 1. Clarity of model design and structure | No description of model design or structure. | Mentions model type (e.g., CLD/SFD) without structural details. | Partial structural description (e.g., variable relationships) . | Full structural description but lacks causal logic or feedback mechanisms. | Clear structure with CLD, SFD, and mathematical equations. | The model structure defines the relationships and organization of elements within the system, influencing its behavior, including responses to external changes and internal interactions. It includes CLD, SFD, feedback loops, policy structures, variables, parameters, delays, equations, tables, graphs, boundary conditions, and subsystems.[2] |
| 1. Detailed description of modeling process | No mention at all. | Briefly mentions steps (e.g., data collection) without methodology. | Describes partial processes but omits stakeholder roles. | Full process description but lacks rationale for key decisions. | Detailed explanation of the process, including stakeholder involvement (e.g., interviews, workshops) and iterative refinement. | Provides reproducible code/toolchain and publicly shares stakeholder feedback records.[1] |
| 1. Comprehensiveness of model validation and verification | No mention at all. | States model is validated but provides no methodology. | Describes single validation method (e.g., historical fit) without multi-scenario coverage. | Multi-dimensional validation (structural + behavioral) but validation data not shared. | Verification and validation procedures adequately described and generally appropriate. | Verification and validation procedures appropriate and transparent validation with third-party peer review or independent replication.[1, 2] |
| 1. Clarity and interpretability of model visualization | No diagrams or visualizations provided. | Basic diagrams provided but are cluttered or unclear. | Readable diagrams but lack annotations for key variables or feedback loops. | Includes CLD/SFD but does not adhere to standard notation. | Diagrams adhere to standard notation and clearly describe model structure | Clear diagrams following SD standards, with dynamic behavior annotations (e.g., oscillations, delays).[1] |
| 1. Clarity and Reliability of Data Sources | No description of data sources or types. | Mentions data sources but omits collection methods or preprocessing. | Describes partial data (e.g., surveys) but ignores bias/missing values. | Full data description but lacks quality assessment. | Transparent sources with cross-validation (e.g., interviews + databases). | Open-source/publicly accessible data with automated cleaning scripts. |
| 1. Present clear model output and results | No mention at all. | Unstructured data (e.g., disorganized tables) without key metrics. | Structured results but no dynamic behavior explanations. | Complete results with sensitivity analysis but unlinked to objectives. | Results directly align with objectives, including dynamic behavior explanations (e.g., role of feedback loops). | Results inform policy recommendations with cross-scenario comparative analysis.[3] |
| 1. Results interpreted and discussed in context | No interpretation or discussion of results in context. | Results are partially interpreted, but key questions (e.g., plausibility, coherence, comparison) are not addressed. | Results are interpreted with attention to precision, plausibility, context, and comparisons (partial coverage). | Results are interpreted and discussed with partial coverage of criteria, but depth is limited. | Results are thoroughly interpreted and discussed, addressing all criteria with clear contextual relevance. | Results are comprehensively analyzed, with detailed comparisons to other studies, robust explanations, and actionable insights for real-world applications.[3] |

**References**

[1] Kenzie E S, Seater M, Wakeland W, et al. System dynamics modeling for cancer prevention and control: A systematic review. PLoS One. 2023;18(12):e0294912. doi:10.1371/journal.pone.0294912.

[2] Chen X, Cai K, Xue Y, et al. Using system dynamics modeling approach to strengthen health systems to combat cancer: a systematic literature review. J Med Econ. 2025;28(1):168-185. doi:10.1080/13696998.2025.2450168.

[3] Fone D, Hollinghurst, S, Temple M, et al. Systematic review of the use and value of computer simulation modelling in population health and health care delivery. J Public Health Med. 2003;25(4):325-335. doi:10.1093/pubmed/fdg075.
